# Synthesis and Characterization of Electrospun Composite Scaffolds Based on Chitosan-Carboxylated Graphene Oxide with Potential Biomedical Applications

**DOI:** 10.3390/ma14102535

**Published:** 2021-05-13

**Authors:** Elena Cojocaru, Jana Ghitman, Elena Iuliana Biru, Gratiela Gradisteanu Pircalabioru, Eugeniu Vasile, Horia Iovu

**Affiliations:** 1 Advanced Polymer Materials Group, University Politehnica of Bucharest, 1-7 Polizu, 011061 Bucharest, Romania; elena.cojocaru3105@upb.ro (E.C.); jana.ghitman@upb.ro (J.G.); iuliana.biru@upb.ro (E.I.B.); 2 Research Institute of the University of Bucharest (ICUB), University of Bucharest, 91-95 Splaiul Independentei, 050095 Bucharest, Romania; gratiela.gradisteanu@icub.unibuc.ro; 3 Department of Science and Engineering of Oxide Materials and Nanomaterials, Faculty of Applied Chemistry and Material Science, University Politehnica of Bucharest, 1-7 Polizu, 011061 Bucharest, Romania; eugeniuvasile@yahoo.com; 4 Academy of Romanian Scientists, 54 Splaiul Independentei, 050094 Bucharest, Romania

**Keywords:** chitosan, carboxyl-modified graphene oxide, composite scaffolds, nanofibrous architecture, cellular viability

## Abstract

This research study reports the development of chitosan/carboxylated graphene oxide (CS/GO-COOH) composite scaffolds with nanofibrous architecture using the electrospinning method. The concept of designed composite fibrous material is based on bringing together the biological properties of CS, mechanical, electrical, and biological characteristics of GO-COOH with the versatility and efficiency of ultra-modern electrospinning techniques. Three different concentrations of GO-COOH were added into a chitosan (CS)-poly(ethylene oxide) (PEO) solution (the ratio between CS/PEO was 3/7 (*w*/*w*)) and were used in the synthesis process of composite scaffolds. The effect of GO-COOH concentration on the spinnability, morphological and mechanical features, wettability, and biological properties of engineered fibrous scaffolds was thoroughly investigated. FTIR results revealed the non-covalent and covalent interactions that could take place between the system’s components. The SEM micrographs highlighted the nanofibrous architecture of scaffolds, and the presence of GO-COOH sheets along the composite CS/GO-COOH nanofibers. The size distribution graphs showed a decreasing trend in the mean diameter of composite nanofibers with the increase in GO-COOH content, from 141.40 nm for CS/PG 0.1% to 119.88 nm for CS/PG 0.5%. The dispersion of GO-COOH led to composite scaffolds with increased elasticity; the Young’s modulus of CS/PG 0.5% (84 ± 4.71 MPa) was 7.5-fold lower as compared to CS/PEO (662 ± 15.18 MPa, *p* < 0.0001). Contact angle measurements showed that both GO-COOH content and crosslinking step influenced the surface wettability of scaffolds, leading to materials with ~1.25-fold higher hydrophobicity. The in vitro cytocompatibility assessment showed that the designed nanofibrous scaffolds showed a reasonable cellular proliferation level after 72 h of contact with the fibroblast cells.

## 1. Introduction

The notable properties of electrospun nanofibrous structures, such as large surface area-to-volume ratio, high degree of porosity and permeability, enhanced stability, and versatility in surface functionality, make them be perceived as important biomaterials with extensive applications in tissue engineering, controlled drug delivery [1] and wound dressing [2].

The chemical and biological features of chitosan (CS) are given by the presence of amino (-NH_2_) and hydroxyl (-OH) groups from the chemical structure. Moreover, owing to its polycationic character, CS can form various stable complexes via hydrogen bonds or electrostatic interactions with a wide range of negatively charged compounds. CS exhibits several inherent properties such as antibacterial and hemostatic activity, bio-adhesion, biocompatibility, and biodegradability as well as a good wound healing potential [3]. Moreover, CS is characterized by processability and versatility in formulation; accordingly, the literature reports a wide number of research studies based on chitosan materials manufactured in various physical forms and combinations using standard casting-out methods (CS-gelatin hybrid porous scaffolds [4], CS-collagen scaffolds crosslinked with genipin [5], CS-based collagen-gelatin composites scaffolds [6], nanocomposite CS films containing graphene oxide-hydroxyapatite-gold [7]) with potential biomedical applications [8,9]. Huang et al. designed CS/gelatin/β-glycerol phosphate hydrogels as carriers for collagenase application in tendon bone healing [10]. In another study, Palma et al. studied the in vivo regenerative potential of CS-based scaffolds after regenerative endodontic procedures (REPs) of immature dog teeth with pulp necrosis and apical periodontitis [11]. According to histological investigations, the use of chitosan scaffolds in REPs did not influenced the formation of new mineralized tissue, but the REPs allowed the development of root walls, increasing bone regeneration.

However, the main drawback of CS is related with the electrospinning difficulties which are associated with its polycationic character, high viscosity and the specific inter- and intra-molecular interactions of CS solution [12].

Therefore, for designing CS fibers, the polymer solution is usually subjected to the electrospinning process in the presence of a hydrophilic synthetic polymer with high molecular weight, such as polyethylene oxide (PEO). PEO can increase the spinnability of CS solution and can contribute to the enhancement of the material’s hydrophilicity [13]. Furthermore, CS-based composite scaffolds with nanofibrous architecture that would better suit the physico-mechanical and biological requirements of living tissue can be designed by insertion of different (bio)macromolecules or other therapeutic and/or reinforcing agents, such as graphene oxide (GO) [14], hydroxyapatite [15], doxorubicin [16] or phospholipids [17]. These agents, even in a small amount, can enhance one or both the therapeutic and mechanical characteristics [18] as well as can improve the surface chemical functionality of the scaffold, a key parameter for the targeted biomedical applications.

The interest and exploitation of graphene oxide (GO) in combination with other (bio)polymers in various fields of bioengineering and medicine are owed to its physico-chemical and biological properties, such as high specific surface area, thermal and electrical conductivities, surface functionality, good antibacterial activity [19] and biocompatibility [20], attachment, adsorption, and recognition capability for different cells and biomolecules (chondrocytes [21], human mesenchymal stem cells [22], osteoblast cells [23], fibroblast cells [24], proteins [25]).

Carboxylated graphene oxide (GO-COOH) represents the carboxyl-modified GO form, characterized by the presence of carboxyl functionalities preponderantly linked on the lateral side of the planar structure of the graphene sheets. GO-COOH presents a more hydrophilic character as compared to graphene, likewise the carboxyl groups contribute to its functionalization with diverse biomolecules. Accordingly, the dispersibility of GO-COOH in water is more satisfactory than that of graphene [26], but it is not enough to form a high stable aqueous dispersion. Therefore, the use of non-ionic surfactants such as Triton X-100 represents a feasible way, very well described in the specialized literature, that can improve the aqueous GO-COOH dispersibility and stability by reducing the non-covalent interactions and the surface tension of graphene sheets in aqueous media [27].

It was shown that the addition of GO within the CS/GO-based composite nanofibrous scaffolds presents a better support for cell proliferation and an increased tensile strength as compared to nanofibrous scaffolds based on CS, for their potential use as artificial cartilage [28]. Furthermore, Liu et al. proved that the mechanical properties and antibacterial activity of CS/PVA nanofibers can be enhanced by the addition of GO [29]. In another study, Sattari and co-workers reported the successful design of a new generation of co-delivery systems based on graphene-g-cyclodextrin/chitosan nanofibers with core-shell architecture as a potent drug delivery system used in tumor inhibition and tissue regeneration [30]. The nanofibers presented a core-shell structure: curcumin; the therapeutic agent was encapsulated within the cyclodextrin-GO core, while to enhance the therapeutic activity of curcumin in the chitosan shell, gallic acid was added. In the biosensors field, Pavinatto and co-workers developed a highly sensitive biosensor based on PVP/CS/reduced-GO electrospun nanofibers for ethinylestradiol electrochemical detection [31], while in another research study, CS/GO/glucose oxidase nanofibers were deposited on the surface of glassy carbon electrode to create a glucose biosensor [32].

Over time, the electrospinning technique has evolved, thus developing a fibrous structure with different configurations and controlled architecture, such as the core-shell structure of scaffolds using coaxial electrospinning. For example, the work of Robinson et al. provided a detailed description of the various methods for obtaining aligned fibers or anisotropic mats, such as post-drawing, near field, gap, or using rotational, centrifugal, or magnetic collecting systems, to control the cellular behavior and mechanical properties [33]. Alishahi and team designed the drug-loaded core-shell nanofibers, using poly(ethylene oxide)/gelatin as shell and poly(vinyl alcohol)/chitosan/glucantime as core, in order to facilitate the release of glucantime to the site of infection, which is produced by cutaneous leishmaniasis [34]. Baldino and co-workers proposed a new technique, called supercritical assisted electrospraying, which involves the addition of supercritical CO_2_ to an initial polymeric solution to decrease its surface tension and viscosity, allowing the design of controlled size micro- and nanoparticles. Then, the same team developed the above-mentioned technology through an extension, this aiming not only to obtain micro- and nanoparticles, but also micro- and nanofibers from polyvinylpyrrolidone (PVP) at high production rates, with a good control over diameter and distribution, using supercritical CO_2_-assisted electrospinning [35].

The literature reports few recent studies in which composite nanofibers based on CS and different amounts of GO are approached, to generate composite nanofibrous scaffolds with potential application in various fields of biomedicine [28,29,30,31,32].

To the best of our knowledge, electrospun composite scaffolds designed by combining CS with GO-COOH, with potential applications in the biomedical field, have not yet been reported in the literature. The CS/GO-COOH composite mixture was approached in a study, in which composite spheres were obtained through sonication, static aging and static solidification, to be used as adsorbent for in situ Cu^2+^ immobilization in soil and to reduce its accumulation in wheat plants [36].

The purpose of this research study was to design chitosan/carboxylated graphene oxide (CS/GO-COOH) composite scaffolds with nanofibrous architecture as biomaterials to be used in tissue engineering. The CS/GO-COOH composite scaffolds with different amounts of GO-COOH were obtained using the electrospinning approach and were further subjected to a crosslinking process in the glutaraldehyde (GA) vapors. The functionality of obtained nanofibrous composite scaffolds as biomaterials was investigated: structurally (FTIR and Raman spectrometry) to confirm the covalent and non-covalent interactions that appear between the system’s components to investigate the exfoliation or intercalation of GO-COOH layers into the CS/PEO polymeric matrix, respectively; morphologically (SEM microscopy) to highlight the nanofibrous architecture of the scaffolds and the presence of GO-COOH sheets along the nanofibers; thermomechanically using DSC and nanoindentation techniques; in addition, in vitro cytocompatibility ((3-(4, 5-dimethylthiazol-2-yl)-2, 5-diphenyltetrazolium bromide) (MTT) assay) and cytotoxicity (lactic dehydrogenase (LDH) assay) of the obtained materials were also studied.

**Figure 1:**
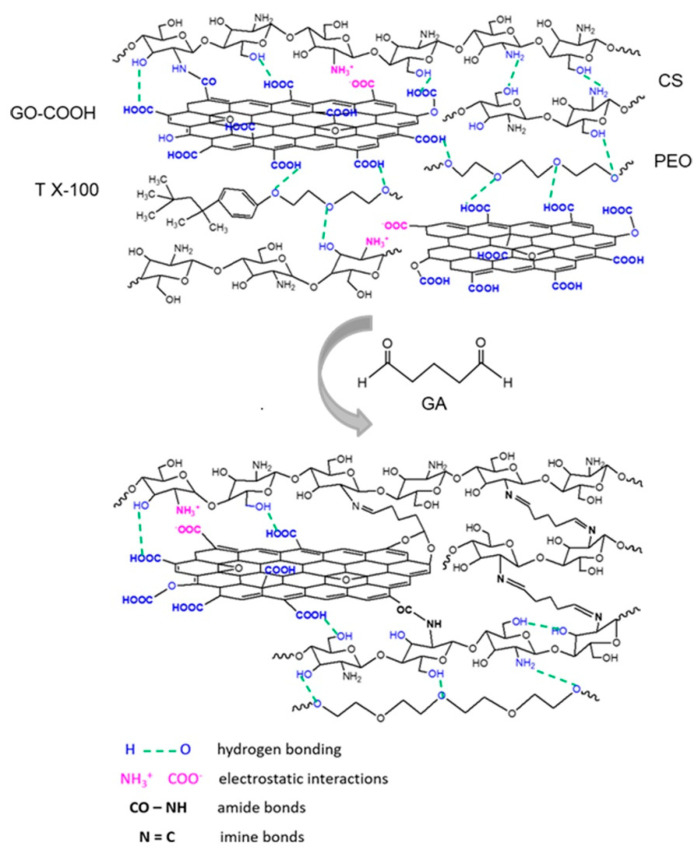
Schematic representation of different types of interactions that can take place between the functionalities of the structures used in the design of composite scaffolds with nanofibrous architecture. Abbreviations: GO-COOH—carboxylated graphene oxide; CS—chitosan; T X-100—Triton X-100; PEO—poly(ethylene oxide); GA—glutaraldehyde.

The advantage of using GO-COOH instead of GO is the enrichment of the system with carboxylic functionalities. The carboxylic groups of GO-COOH can generate both non-covalent interactions (either hydrogen bonds or electrostatic interactions [37]) with the hydroxyl (-OH) and amino (-NH_2_) groups of CS, polyether groups from the PEO and Triton X-100 structure; and covalent interactions (amide bonds) with amino (-NH_2_) groups presented on the polycation chains structure. At the same time, the crosslinking agent, GA, will interact through aldehyde functional groups not only with the primary amine from the CS structure, forming covalent imine (C=N) bonds, but also, it is expected to interact with hydroxyl groups presented in the CS chemical structure (as was shown in FTIR spectra) generating acetal bonds [38], improving the stability of the composite scaffolds (Figure 1).

## 2. Materials and Methods

### 2.1. Materials

Chitosan (CS) with medium molecular weight (Mw) and 75–85% degree of deacetylation, poly(ethylene oxide) PEO with Mw of 600,000 Da, 4-(1,1,3,3-Tetramethylbutyl) phenyl-polyethylene glycol Triton X-100, acetic acid CH_3_-COOH with 99.8–100.5% purity and glutaraldehyde grade I (GA), and 50% aqueous solution were obtained from Sigma-Aldrich (Sigma-Aldrich Chemie GmbH, Germany). Commercial carboxyl-modified graphene oxide (GO-COOH) with a concentration of carboxylic groups of 0.7 mmol COOH/g was provided by NanoInnova Technologies (NIT, Toledo, Spain). Only ultra-pure water from the Milli-Q Plus system (Millipore, MA, USA) was employed in all experiments.

### 2.2. Preparation of electrospinning solutions

The 3% (*w*/*v*) CS solution was obtained by dissolving the required amount of CS in 5M (*v*/*v*) acetic acid aqueous solution under heating at 60 °C and stirring conditions for 6 h, to ensure the complete dissolution, followed by centrifugation of the solution to remove the undissolved chitosan sediments. The 3% (*w*/*v*) PEO solution was prepared in ultra-pure water, under heating at 80 °C and stirring conditions, until PEO was completely dissolved. Then, the two polymer solutions were mixed in a 3/7 CS/PEO (*w*/*w*) ratio and the mixture was subjected to electrospinning process after complete homogenization in order to produce CS-based electrospun scaffolds.

To engineer the electrospun composite scaffolds based on CS/GO-COOH, different concentrations of GO-COOH (Table 1) in 5M (*v*/*v*) acetic acid aqueous solutions and 1% (*w*/*v*) Triton X-100 under ultrasonication were prepared. Then, to the obtained dispersions, the specific amounts of CS and PEO to maintain the 3/7 ratio were added. Prior to being electrospun, the systems were subjected to homogenization until complete polymer solubilization.

### 2.3. Electrospinning Process and Parameters

The electrospinning process was performed using a climate-controlled electrospinning equipment (IME Technologies, Waalre, The Netherlands). A volume of 3 mL from each previously prepared CS-based solution or composite dispersion were loaded into a horizontal syringe provided with a needle having 0.6 mm in inner diameter through which the solution was pushed with the help of a syringe pump. The fibrous meshes were deposited on the grounded rotating collector, covered with aluminum foil. The tip-collector distance was 15 cm, and the intensity of applied voltage was set between 10 and 20 kV. All samples were electrospun at an ambient temperature of 25 °C and a relative humidity between 30 and 45%.

### 2.4. Crosslinking of Electrospun Nanofibrous Scaffolds

The obtained electrospun meshes were cut into rectangular strips with a size of 30 mm × 10 mm and placed in a covered recipient containing 50% GA aqueous solution. The scaffolds were suspended in GA vapors for 4 days; then, they were immersed in ultra-pure water for 5 days to remove the residual GA. Finally, the crosslinked electrospun nanofibrous scaffolds were dried in an oven at 37 °C for 2 h.

### 2.5. Characterization Methods

#### 2.5.1. Hydrodynamic Diameter, Polydispersity Index and Diffusion

Hydrodynamic diameter (d), polydispersity index (PdI) and diffusion (D) were investigated through the Dynamic Light Scattering (DLS) technique using Nano Zetasizer ZS equipment (Malvern Panalytical, Worcestershire, UK). For DLS measurements, systems consisting of PEO, CS, GO-COOH, CS/PEO, and CS/PEO/GO-COOH at a concentration of 1 mg/mL in 5M acetic acid solution were prepared. The acetic acid solution was filtered through a 0.2 μm syringe filter before use. The hydrodynamic measurements were carried out at room temperature (25 °C). The results represent the average of 3 measurements each being run in 12 successive cycles.

#### 2.5.2. Structural Characterization

Fourier Transform Infrared (FTIR) spectra were registered on a Bruker Vertex 70 spectrometer (Bruker, Billerica, MA, USA), provided with an attenuated total reflectance (ATR) component. For all used materials and the obtained nanofibrous scaffolds, the FTIR spectra were recorded in the ATR mode, in a wave number range of 4000–600 cm^−1^, at a resolution of 4 cm^−1^, and 32 scans for each material.

Raman spectra were registered on a Renishaw inVia Raman microscope system (Renishaw, Brno-Černovic, Czech Republic) in the range of 100–3200 cm^−1^. The wavelength of the excitation laser used was 473 nm, with a laser power of about 50%, and 3 accumulations for 10 s were recorded. The laser beam was concentrated with the 100 × objective of the microscope on the surface of the sample.

#### 2.5.3. Morphological Characterization

The morphological features of all un-crosslinked nanofibrous scaffolds were examined by Quanta Inspect F50 Scanning Electron Microscopy (SEM) (FEI, Hillsboro, OR, USA) with a field emission electron gun with 1.2 nm resolution. The dried materials were sputter-covered with a thin gold sheet and their morphology was investigated. The mean diameters of the nanofibers were calculated by averaging the diameter of 100 random fibers.

#### 2.5.4. Mechanical Analyses

The mechanical properties at the nano-level, such as Young’s modulus and hardness, of all the crosslinked nanofibrous scaffolds were studied by the nanoindentation technique using the Nano Indenter Agilent G200 instrument, equipped with an XP head (Keysight Technologies, Santa Rosa, CA, USA). The electrospun mats with thickness in the range of 380–426 μm were fixed on a sample holder using an adhesive double strip for the NanoVision stage. The measurements were performed using the Express Test to a Displacement Large Table method from the NanoSuite software. For each sample, 100 indents at a 50 μm distance were made using a Berkovich diamond tip with a 20 nm radius. The nanoindentation depth was 300 nm, with a strain rate of 0.05/sec, setting the Poisson ratio at 0.4. The experiments were performed in triplicate.

#### 2.5.5. DSC Investigations

Differential scanning calorimetry (DSC) examinations were performed on a Netzsch DSC 204 F1 Phoenix (Netzsch -Gerätebau GmbH, Selb, Germany) calorimeter using a constant nitrogen flow of 20 mL/min. The analyses were carried out with a heating rate of 10°/min, in the ±20–300 °C temperature range. Each sample containing a 7–8 mg nanofibrous scaffold was placed in a hermetically sealed aluminum pan. The analysis was replicated in triplicate.

#### 2.5.6. Wettability Investigations

Information about the wettability of all un-crosslinked and crosslinked electrospun scaffolds was obtained using the Drop Shape Analyzer-DSA100 from Krüss Scientific GmbH (Hamburg, Germany) and the sessile drop method. The effect of the crosslinking step upon the hydrophobicity of CS-based scaffolds or composite scaffolds with nanofibrous architecture was evaluated by static water contact angle measurements at room temperature. The shape of the deionized water drop on the sample surface was recorded with a CF03 digital camera for 5 s after deposition of the droplet with a volume of 2 μL water. The water contact angle was determined using the Advance software and represents the average of three measurements for each sample. The results were revealed using the Young–Laplace equation.

#### 2.5.7. In Vitro Degradation

The in vitro degradation of un-crosslinked and crosslinked nanofibrous scaffolds was studied by incubating the lyophilized materials in phosphate-buffered saline solution (PBS, pH 7.4) at 37 °C for 96 h. At a predetermined period of time (12, 24, 48, 72, and 96 h), the samples were removed from the medium, lyophilized, and weighed. The experiments were performed in triplicate and the in vitro degradation rate was calculated using Equation (1):

(1) $$D\;(\%)=\frac{W_{0}-\;W_{d}}{W_{0}}\;\times \;100$$

where: D—mass loss expressed in percentage (%); w_0_—initial weight; w_d_—dry weight.

#### 2.5.8. In Vitro Cytocompatibility and Cytotoxicity

The in vitro cytocompatibility was accomplished by incubating the nanofibrous scaffolds with fibroblasts for 24 and 72 h, in DMEM medium containing 10% fetal bovine serum, supplemented with penicillin and streptomycin. The cells were seeded in 96-well plates with a density of 1 × 105/mL and incubated for 24 h at 37 °C in a humidified atmosphere of 95% air and 5% CO_2_ to allow cell attachment. The MTT viability assay allows the quantitative evaluation of living cells in culture; the MTT compound is permeable to living cell membranes. Then, each sample was incubated into 1 mL MTT solution for 4 h at 37 °C and 5% CO_2_. The resulted formazan produced by the metabolically active cells was measured using a spectrophotometer (Flex Station 3), at a wavelength of 550 nm.

The cytotoxic effect was evaluated using the LDH assay, which indicates the number of dead cells in the culture. Cells without an integral membrane release into medium the LDH containing-cytoplasm. The culture media were mixed with the components of the Tox-7-KT kit, according to the manufacturer’s instructions and incubated in the dark for 20 min. The resulted solution can be read spectrophotometrically at 490 nm. The cell culture without scaffolds was used as a control. The experiment was performed in triplicate.

#### 2.5.9. Statistical Analyses

The data are expressed as mean ± SD. The significance of differences was evaluated by the ANOVA test and it was considered significant if *p* < 0.05.

## 3. Results and Discussions

Although CS is a versatile biopolymer widely used in various biomedical fields, certain limiting factors such as its polycationic character, high viscosity, and the rigidity of polymeric chains affect the electrospinning process. To overcome these limitations, CS was dissolved in acid aqueous medium in order to protonate the amino groups from the polymer chain. The synthetic PEO was employed to further reduce the surface tension of the obtained polycation solution [39], to improve the electrospinning properties and to obtain fibrous scaffolds with optimal morphological features.

### 3.1. Hydrodynamic Studies

The various types of interactions that can take place between the functionalities of the components from the system play a role in defining the integrity and stability of designed material. These interactions can be investigated by monitoring the hydrodynamic characteristics of components involved in that system [40]. The hydrodynamic characteristics d (hydrodynamic diameter), PdI (polydispersity index), and D (diffusion) are presented in Table 2.

Although DLS is extensively used to determine the colloidal characteristics of spherical particles which are undergoing Brownian motion, it can also be applied for qualitative information in other types of colloidal systems [41]. A consistent difference between the hydrodynamic characteristics of colloidal systems consisting of raw materials, mainly attributed to their chemistry, can be observed. The relatively large particles size distribution (d = 890.00 ± 35.73; PdI = 0.66 ± 0.05) of suspended GO-COOH sheets in aqueous acidic medium can be explained by its non-spherical shape supplemented with some re-aggregation phenomena, in corroboration with the literature [42]. In the case of multicomponent CS/PEO and CS/PG 0.2% systems, it appears that a combination of processes (e.g., electrostatic interactions, and hydrogen bonding effects) which take place between the components, determined the modification of their conformation, influencing the diffusion and hydrodynamic features [40].

According to the literature, the diffusion coefficient of a colloid is inversely proportional to its diameter and directly proportional to its mobility [43]. The higher value of diffusion registered for CS/PEO colloids as compared to CS/PG 0.2% systems, which registered a higher diffusion as compared to CS dispersion (presenting the lowest D value compared to the larger agglomerates), may suggest the occurrence of numerous intermolecular interactions between the components of the system.

### 3.2. FTIR Studies

**Figure 2:**
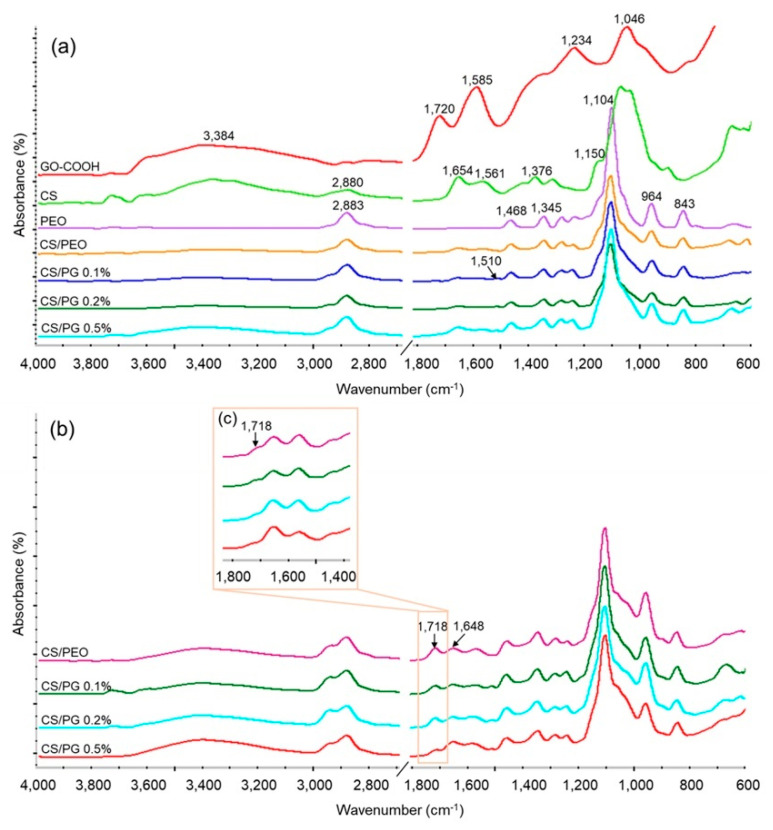
ATR-FTIR spectra of (**a**) raw materials and all un-crosslinked electrospun scaffolds; (**b**) unwashed crosslinked samples (characteristic free-aldehydic peak); and (**c**) 5 days washed crosslinked nanofibrous scaffolds.

The structure of composite electrospun scaffolds as well as the new covalent bonds which were formed after the crosslinking step were investigated by FTIR spectrometry. The FTIR spectra recorded for raw materials, un-crosslinked and crosslinked electrospun CS/PEO scaffolds, or CS/PG composite scaffolds with different GO-COOH content are shown in Figure 2a–c.

The FTIR spectrum of GO-COOH showed five characteristic absorption peaks located at: 3384 cm^−1^ attributed to the stretching vibration of the hydroxyl group (-OH); 1720 cm^−1^ ascribed to stretching vibration of carbonyl bond (C=O) from the carboxyl group; 1585 cm^−1^ corresponding to the stretching vibration of C=C from the planar structure of graphene; 1234 cm^−1^ representing the stretching vibration of C–O from the epoxy group; and 1046 cm^−1^ attributed to the stretching vibration of C–OH from the GO-COOH groups [44].

The FTIR spectrum recorded for CS exhibited the following characteristic absorption bands: a large band in the region 3400–3200 cm^−1^ that corresponded to the stretching vibration of the hydroxyl (-OH) and amine (-NH_2_) groups. The peaks at around 2880 cm^−1^, 1654 cm^−1^ and 1561 cm^−1^ were attributed to the symmetric and asymmetric stretching vibration of the -CH- group, the stretching vibration of the carbonyl bond (C=O) from amide I and the bending vibration of the amino group (NH_2_) from amide II [45] presented in the residual N-acetyl groups from chitosan’s chemical structure. The absorption band at 1376 cm^−1^ corresponded to CH_2_ bending vibration. The peak at 1150 cm^−1^ was attributed to the asymmetric stretching vibration of the C-O-C bridge from the glycosidic linkage from the CS backbone structure [46].

In the FTIR spectrum of PEO, the characteristic absorption peaks at 2883 cm^−1^, 1468 cm^−1^, and 1345 cm^−1^ from the symmetric -CH- stretching and -CH_2_- bending vibrations, respectively, were noted. The sharp peaks at 1104 cm^−1^, 964 cm^−1^ and 843 cm^−1^ were attributed to the stretching vibrations of the ether (C-O-C) and -CH_2_-CO groups from the PEO chemical structure [47].

In the FTIR spectra recorded for the CS/PEO-based scaffold and CS/PG composite scaffolds with nanofibrous architecture, all characteristic absorption bands from both CS and PEO chemical structure were identified, demonstrating the successful combination of polymers into the nanofibers. It is worth mentioning that a new peak at 1510 cm^−1^ was identified in all spectra of CS/PG electrospun composite scaffolds, which can be assigned to the combination of the C–N stretching with CH–N bending of amide II, corresponding to amides formed between CS and GO-COOH during mixing [48].

The FTIR spectra of all unwashed and 5 days washed crosslinked scaffolds are shown in Figure 2b,c. It is important to mention the presence of a new strong peak at around 1718 cm^−1^ in the FTIR patterns recorded for unwashed samples (Figure 2b) and its absence in the spectra recorded for washed nanofibrous scaffolds (Figure 2c). This peak was attributed to the stretching vibration of the C=O group of free-aldehydic bonds from the GA structure [49] and its disappearance demonstrated the efficient elimination of unreacted residual GA from the scaffold’s composition. Further, the crosslinking process takes place between the amino groups of CS and aldehyde groups of GA by a Schiff base reaction [50], leading to a new imine (C=N) bond which appeared in FTIR spectra at around 1648 cm^−1^.

### 3.3. Raman Spectrometry Results

**Figure 3:**
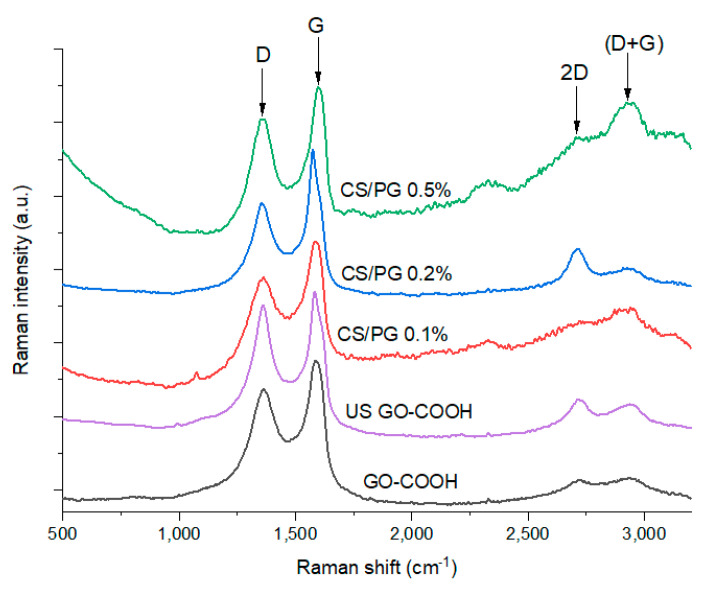
Raman spectra of GO-COOH and GO-COOH subjected to ultrasonication (US) treatment and CS/PG composite nanofibrous scaffolds with varying GO-COOH content.

Raman spectrometry remains an important tool for structural investigation of the GO-based materials as it offers important information regarding the chemical modification of the GO surface. As presented in Figure 3, in both GO-COOH raw material and CS/PG synthetized nanofibrous scaffolds, the characteristic Raman signals of the GO were identified, all spectra presenting the typical intense D (~1360 cm^−1^) and G (~1590 cm^−1^) bands [51]. The D band was used in this study as an indicator for further modifications of the GO-COOH structure with CS and PEO as it acknowledges the presence of sp^3^ carbon atoms formed as a consequence of GO-COOH surface modification and its distribution within the polymer nanofibers. The G band arises from the sp^2^ breathing mode of the graphene aromatic layers, indicating the presence of C=C bonds being characteristic to sp^2^ hybridized carbon nanomaterials. Moreover, this signal can be correlated to the C=C bond vibrations that occurred at 1585 cm^−1^ from the FTIR spectra, which correspond to the graphene planar structure based on aromatic rings. As a measure of structure distortions suffered by GO-COOH along the process, the ratio between the intensity of the D band and the intensity of the G band (I_D_/I_G_) was calculated (Table 3).

The increased I_D_/I_G_ ratio value of ultrasonicated GO-COOH, as compared to the GO-COOH, can be attributed to the creation of more defects in the GO-COOH structure, caused by the exfoliation process.

By comparing the Raman spectra of the CS/PG composite scaffolds with the spectrum of US GO-COOH, it was clearly observed that important structural modifications occurred after the synthesis of the nanofibers as the GO-COOH content was increased. Thus, the I_D_/I_G_ ratio was significantly increased from 0.88 to 0.92 and 0.93 subsequently due to the interactions between GO-COOH and CS/PEO chains when an amount of 0.2 wt.% and 0.1 wt.% GO-COOH was used. As expected, the functionalities of CS and PEO chains play a major role in the dislocation of the GO-COOH layers as a result of the formation of hydrogen bonds or electrostatic interactions that took place between the components, leading to a higher disordered GO-COOH layers with a more significant sp^3^ content compared to the raw material. Furthermore, the addition of 0.5 wt.% GO-COOH in the nanofiber composite led to a significant decrease in I_D_/I_G_ ratio as a result of a lower content of defects in the GO-COOH structure, due to the lower dispersion of the GO-COOH within the polymeric matrix CS/PEO. This fact may be generated by the agglomeration tendency of GO-COOH layers at higher concentration, caused by π–π* stacking interactions between GO-COOH layers, thus re-aggregation of the GO-COOH sheets may take place.

The presence of the 2D signal (~2700 cm^−1^) that characterizes the arrangement and number of GO-COOH layers was clearly noticed in the case of the investigated materials along the (D + G) combination peak at ~2900 cm^−1^, meaning that structural arrangement modifications occurred during the synthesis. In the case of the CS/PG 0.1% scaffold, the broad shape of the 2D band suggests the existence of multiple GO-COOH layers stacked into the polymer matrix. On the contrary, the presence of a sharper 2D band from the CS/PG 0.2% composite spectrum indicates a better exfoliation of GO-COOH agglomerates [52] due to the multiple interactions between PEO (-O-) and CS (-OH, -NH_2_) functionalities and -COOH groups of GO-COOH sheets enhancing the distance between GO sheets. The more pronounced (D + G) combination peak in the spectra of CS/PG 0.1% and CS/PG 0.5% supports the presence of intercalated structures compared to CS/PG 0.2% due to the stacking tendency of GO-COOH layers.

### 3.4. Morphology Investigation

The morphological characteristics of a material represent one of the key parameters that define its potential application in various biomedical fields. The materials with nanofibrous architecture as compared to simple/porous materials are endowed with several superior features such as: structural support, optimal conditions for cells attachment, proliferation and differentiation, as well as good bioactivity attributed to the large surface-to-volume ratio, porous construction with good pore interconnectivity and architectural resemblance to that of the extracellular matrix [53].

**Figure 4:**
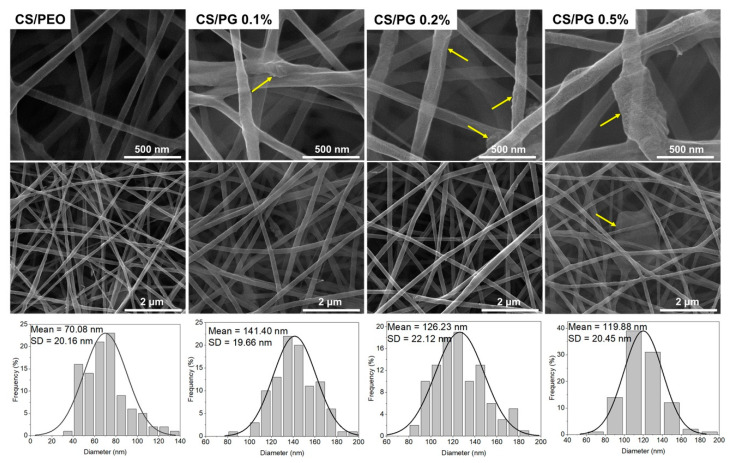
SEM images and the corresponding size distribution graphs of CS/PEO electrospun scaffolds as well as CS/PG 0.1%, CS/PG 0.2%, and CS/PG 0.5% composite scaffolds with nanofibrous architecture (the images are presented at 50,000×, 200,000× magnification).

The microstructure of CS/PEO-based electrospun scaffolds and composite CS/PG nanofibrous scaffolds with different amounts of GO-COOH (CS/PG 0.1%, 0.2%, and 0.5%, respectively) was investigated by SEM microscopy. The collected SEM images with the corresponding size distribution graphs are presented in Figure 4.

The SEM images highlighted a uniform, smooth, continuous, and bead-free nanofibrous structure of the CS/PEO scaffold. Unlike CS/PEO architecture, CS/PG composite scaffolds showed continuous nanofibers with irregular surfaces. As a result of polymer (CS/PEO)-GO-COOH interactions, the GO-COOH sheets (marked with yellow arrows in SEM images) can be either entrapped into the nanofiber structure or may be disposed along their surface, changing the surface morphology of composites. Moreover, the mean diameter of composite nanofibers was influenced by the amount of GO-COOH distributed in the scaffolds. A decreasing trend was noted in the mean diameter of composite nanofibers as GO-COOH content increased. According to the size distribution graphs, the mean diameters of the composite nanofibrous scaffolds were 141.40 nm for CS/PG 0.1%, 126.23 nm for CS/PG 0.2% and 119.88 nm for CS/PG 0.5%, respectively. These changes can be attributed to the electrical conductivity of the electrospinning system which increased with the rising amount of GO-COOH [30]; at the same time, the conductivity is inversely proportional to the nanofiber diameter [29].

Likewise, the non-uniform distribution and the tendency of GO-COOH to agglomerate at a higher concentration were also noticed, in concordance with Raman investigations (CS/PG 0.5% composite scaffolds) and can be attributed to graphene hydrophobicity, van der Waals attractive interactions [54], and non-covalent π–π* stacking interactions [55]. The cluster obstructs the dispersion of the GO-COOH layers through the polymeric matrix, unlike its uniform dispersion at low GO-COOH concentrations (CS/PG 0.1% and 0.2% samples).

### 3.5. Mechanical Features Investigated by Nanoindentation

**Figure 5:**
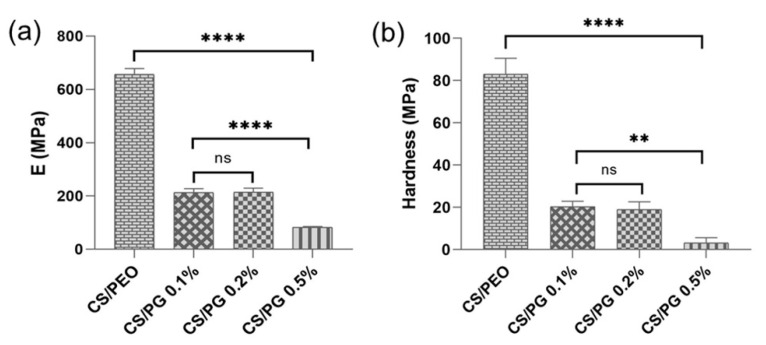
Nano-mechanical characteristics expressed by (**a**) Young’s modulus and (**b**) hardness of investigated nanofibrous structures; ns *p* > 0.5, ** *p* < 0.005, **** *p* < 0.0001.

Nanoindentation is a versatile, ultra-modern and widely used technique in studying the mechanical properties of materials at the nano-level. The impact of GO-COOH on the mechanical features of all crosslinked nanofibrous scaffolds was evaluated by determining and comparing both the Young’s modulus (E) and hardness (H) of each sample (Figure 5).

According to the registered mechanical features (E and H) of nanofibrous scaffolds, it is obvious that the structural modifications that occurred with the addition of GO-COOH were also reflected on the mechanical properties of CS/PG composite scaffolds.

The CS/PEO nanofibrous scaffold was found to be the hardest material with the E and H values of 662 MPa and 82 MPa, respectively. A decreasing trend of both the elastic modulus and hardness compared to an increase in elasticity of the CS/PG composite nanofibrous scaffolds with the increase in GO-COOH amount was noted. This behavior could be attributed to the non-covalent interactions such as hydrogen bonds formed between the GO-COOH and functionalities of CS, and PEO polymers, which may hinder the degree of interactions between the polymer chains, thus increasing the elasticity of composite materials [56]. On the other hand, the agglomeration phenomena of GO-COOH layers, as was clearly observed in Raman and SEM investigations in the case of CS/PG 0.5% sample, can behave as weak points that disturb the structural regularity of the CS/PEO polymeric matrix, leading to the fragility of the nanofibrous scaffolds and decreasing their mechanical properties [57].

### 3.6. DSC Tests

**Figure 6:**
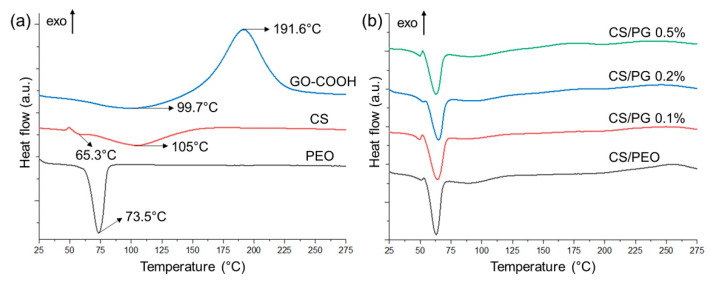
DSC curves of (**a**) raw materials (PEO, CS, GO-COOH); (**b**) crosslinked nanofibrous scaffolds.

The thermal behavior of all crosslinked electrospun CS/PEO, composite CS/PG scaffolds and raw materials was investigated by DSC (Figure 6a,b and Table 4).

The DSC curve of GO-COOH is characterized by a broad endothermic peak at 99.7 °C which is attributed to the dehydration process and a pronounced exothermic peak at 191.6 °C that indicates the de-oxygenation of GO-COOH (the degradation of -COOH and -OH functionalities from the surface of GO-COOH sheets) [58]. The CS’ curve is characterized by two different degradation phases. The first endothermic peak at 65.3 °C can be assigned to the moisture loss related to the hydrophilic groups from the CS structure, while the second broad endothermic peak at around 105 °C may be attributed to the thermal decomposition of the CS structure [59]. In the case of the DSC curve registered for PEO, the sharp peak at 73.5 °C is assigned to the polymer’s T_m_ [60].

Regarding the crosslinked electrospun scaffolds (Figure 6b), it seems that the amount of GO-COOH dispersed within the polymeric matrix did not impact the T_m_ values in a significant way (the registered T_m_ values were in the range of 63.00 to 65.10 °C). The dispersion of 0.1 wt.% and 0.2 wt.% GO-COOH with respect to the polymer matrix determined a slight shift of the T_m_ to high values (64.10 °C and 65.10 °C), while when the GO-COOH concentration was increased to 0.5 wt.% (CS/PG 0.5% composite scaffolds), the T_m_ value was slightly shifted to 63.20 °C. The same effect was also reported in the work of C-L. Huang and co-workers, where with increasing the graphene nanosheet concentration, a slight decrease in T_m_ of the composite PTT nanofibers was observed [61]. Probably, this decrease is due to the formation of GO-COOH agglomerates, as seen in SEM and Raman analyses, which can alter the polymeric matrix structure, determining a decrease in thermal stability. However, the melting enthalpy (ΔH_m_) of the CS/PG composite scaffolds is higher as compared to the CS/PEO samples and slightly increased with the rise in GO-COOH concentration

### 3.7. Wettability Properties

**Figure 7:**
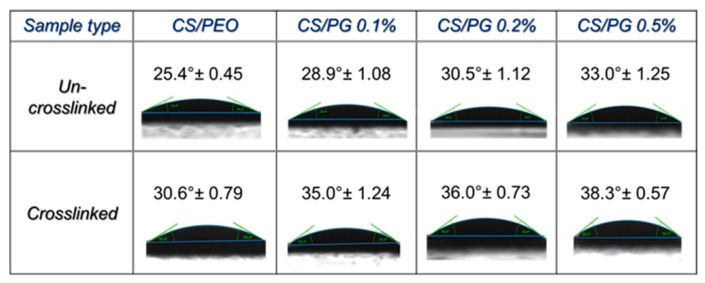
Water contact angle of all engineered nanofibrous scaffolds.

The wettability properties of designed CS/PEO or CS/PG composite nanofibrous scaffolds may be influenced by the amount of GO-COOH dispersed into the polymeric system and the crosslinking process. The wettability features of all investigated un-crosslinked and crosslinked nanofibrous samples expressed as water contact angle are presented in Figure 7.

Generally, according to the contact angle results, the surfaces of all analyzed nanofibrous scaffolds presented a hydrophilic character. A slight increasing trend in the surface hydrophobicity of composite scaffolds (the water contact angle increased) with an increasing amount of GO-COOH, in both un-crosslinked and crosslinked nanofibrous scaffolds, can be observed.

The contact angle value of the CS/PEO nanofibrous scaffold was found to be 25.4° due to the hydrophilic functional groups (hydroxyl, amino, ether) of both polymers. The dispersion of 0.1 wt.% GO-COOH shifted this value to 28.9°, and if a high amount of GO-COOH was used (CS/PG 0.5% nanofibrous scaffolds), an increase of approximately 15% of the contact angle (33.0°) was noted and was correlated to the intrinsic hydrophobicity of the graphene layers [29]. Similar to the GO-COOH effect, the crosslinking process increased the surface hydrophobicity (expressed as contact angle value) of all nanofibrous scaffolds by approximately 15–20%.

However, the registered contact angle value suggests the sufficient hydrophilic character of designed nanofibrous scaffolds for potential biomedical applications.

### 3.8. In Vitro Degradation Studies

**Figure 8:**
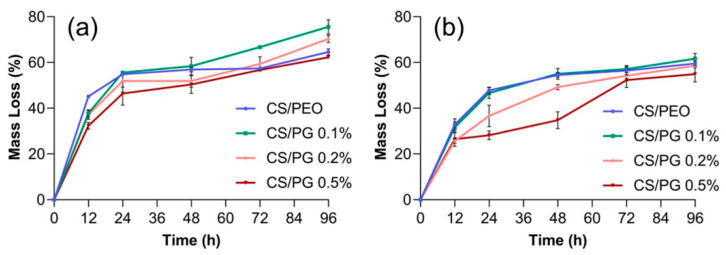
In vitro degradation rate of (**a**) un-crosslinked and (**b**) crosslinked nanofibrous scaffolds in PBS solution after 12, 24, 48, 72, and 96 h.

The in vitro degradation rate of un-crosslinked and crosslinked nanofibrous scaffolds containing different concentrations of GO-COOH, after 12, 24, 48, 72, and 96 h, are presented in Figure 8.

The mass loss of nanofibrous scaffolds is mainly influenced by the chemical crosslinking and the nature of materials embedded in the scaffold. As was expected, the crosslinking step in the presence of GA vapors improved the stability of fibrous scaffolds in the PBS solution, through the creation of new covalent bonds, such as imine bonds, between the constituents of the polymeric matrix and GA. It can be observed that the un-crosslinked materials registered a higher mass loss compared to the crosslinked ones, suggesting the efficiency of the crosslinking step. After 12 h of incubation in PBS solution, the percent of mass loss of the un-crosslinked CS/PEO scaffold was ~15% higher as compared to that of the crosslinked scaffold. The degradation rate of the un-crosslinked CS/PG composite scaffolds was also higher as compared to their crosslinked counterparts; the mass loss values of un-crosslinked and crosslinked CS/PG 0.5% after 24 h were 50% and 28.57%, which increase to 53.13% and 32.14%, respectively, after 48 h, and reached 62.50% and 52.50% at the end of the experiment.

The dispersion of GO-COOH slightly decelerated the degradation rate of composite fibrous scaffolds. The mass loss decreased with rising the GO-COOH concentration and this trend is more pronounced in the case of crosslinked fibrous scaffolds.

### 3.9. In Vitro Cytocompatibility and Cytotoxicity Assessment

**Figure 9:**
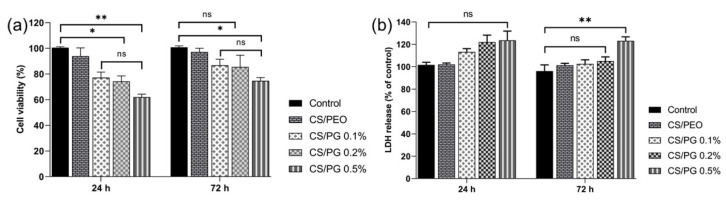
In vitro cytocompatibility of all electrospun scaffolds on fibroblast cells, after 24 and 72 h; (**a**) MTT assay; controls at 24 and 72 h are considered as 100% (ns *p* < 0.5, * *p* < 0.05, ** *p* < 0.005); and (**b**) LDH assay (ns *p* < 0.1, ** *p* < 0.01).

The in vitro cytocompatibility assessment of all designed electrospun scaffolds with nanofibrous architecture was performed on fibroblast cells after 24 and 72 h, using MTT viability and LDH cytotoxicity assays (Figure 9).

After 24 h, the cellular viability measured in the CS/PG composite materials was lower as compared to that of the control; the number of living cells decreased with the increasing amount of GO-COOH. After 24 h of exposure, the lowest cellular viability was registered in the CS/PG 0.5% sample (*p* < 0.005). Major improvement in cellular viability and a good proliferation potential were observed in all investigated materials after 72 h of fibroblast incubation, as compared to the results obtained after 24 h. Then, a slight decreasing trend in cell viability and proliferation potential was observed when the amount of GO-COOH was increased, the lowest level of fibroblast activity being registered for CS/PG 0.5% (*p* < 0.05). At the same time, the LDH results showed no important differences in the cytotoxic responses registered for the control and CS/PEO, CS/PG 0.1% and CS/PG 0.2% scaffolds after 24 h or 72 h of incubation. At the same time, after 72 h, the cytotoxic response of fibroblasts incubated in the presence of CS/PG 0.5% was visibly higher as compared to the control (*p* < 0.01) and could be correlated with the GO-COOH agglomerates (as was observed in Raman), which may affect the cell development.

## 4. Conclusions

The composite scaffolds with nanofibrous architecture based on natural/synthetic polymeric matrix (CS and PEO) and GO-COOH were engineered using the electrospinning technique. The effect of GO-COOH on the structural, morphological and wettability characteristics, as well as on the mechanical, thermal, and in vitro biological behavior was investigated in detail.

Non-covalent interactions (such as electrostatic and hydrogen bonding) which occurred between the components were observed in DLS, while the new covalent bonds (imine and ether) formed after the crosslinking step were seen in FTIR. Raman investigation emphasized the uniform dispersion of GO-COOH within the CS/PG 0.1% and CS/PG 0.2% composite scaffolds. SEM micrographs highlighted the uniform and bead-free structure of the CS/PEO nanofiber and the irregular surfaces with GO-COOH sheets uniformly dispersed along the composite nanofibers, accomplished with a decreasing trend in mean diameter with the increase in GO-COOH content (from 141 nm for CS/PG 0.1% to 119 nm for CS/PG 0.5%).

As was expected, the hydrophobic properties of GO-COOH sheets and the crosslinking step decreased the surface wettability and the degradation rate of materials. Then, the dispersion of GO-COOH influenced the mechanical properties of the composite nanofibrous scaffolds by decreasing both the Young’s modulus and hardness.

According to the in vitro cytocompatibility and cytotoxicity assessment, the fibroblast cells culture showed a reasonable proliferation level after 72 h of incubation in the presence of CS/PG 0.1% and CS/PG 0.2% nanofibrous scaffolds. However, the decrease in cell viability and a slight increase in cell cytotoxicity observed at high GO-COOH concentration (CS/PG 0.5%) may be attributed to some agglomeration phenomena of GO-COOH sheets, as observed in Raman and SEM analyses.

Although, the GO-COOH suspension was subjected to ultrasonication in the presence of Triton X-100 in order to improve the dispersibility, when a high concentration was used, some agglomeration of GO-COOH sheets was still observed, which lowered the general properties of composite CS/PG 0.5% nanofibrous scaffolds. Considering this drawback, further investigations will be directed towards improving the dispersibility of the GO-COOH sheets, when a high concentration is used in a precursor mixture as well as in the electrospun scaffolds; subsequently, more comprehensive in vitro biological investigations need to be performed.

## Figures and Tables

**Table 1:** The composition of all electrospun scaffolds.

Sample	V (mL)	CS/PEO (*w*/*w*) Ratio	c(GO-COOH) wt.%	c (Triton X-100) wt.%
CS/PEO	5	3/7	0	1
CS/PG 0.1%			0.1	
CS/PG 0.2%			0.2	
CS/PG 0.5%			0.5	

**Table 2:** The hydrodynamic characteristics of raw materials and CS/PEO and CS/PG 0.2% dispersions.

Sample	d (nm)	PdI	D (µm^2^/s)
CS	1108.00 ± 40.31	0.38 ± 0.01	0.45 ± 0.01
PEO	86.83 ± 16.55	0.30 ± 0.01	6.78 ± 0.18
GO-COOH	890.00 ± 35.73	0.66 ± 0.05	0.53 ± 0.02
CS/PEO	76.66 ± 2.95	0.45 ± 0.03	1.25 ± 0.03
CS/PG 0.2%	836.50 ± 13.42	0.40 ± 0.01	1.16 ± 0.01

**Table 3:** The I_D_/I_G_ ratio of GO-COOH and GO-COOH subjected to ultrasonication (US) treatment, and CS/PG composite nanofibrous scaffolds from Raman investigation.

Sample	ν_D_ (cm^−1^)	ν_G_ (cm^−1^)	I_D_/I_G_ (473 nm laser)
GO-COOH	1360	1592	0.80
US GO-COOH	1358	1589	0.88
CS/PG 0.1%	1354	1589	0.93
CS/PG 0.2%	1356	1578	0.92
CS/PG 0.5%	1354	1601	0.84

**Table 4:** Melting temperature (T_m_) and melting enthalpy (ΔH_m_) of the crosslinked nanofibrous scaffolds.

Sample	T_m_ (°C)	ΔH_m_ (J/g)
CS/PEO	63.00 ± 1.26	53.26 ± 1.06
CS/PG 0.1%	64.10 ± 2.56	58.51 ± 2.34
CS/PG 0.2%	65.10 ± 1.75	59.33 ± 1.60
CS/PG 0.5%	63.20 ± 2.84	61.67 ± 2.77

## Data Availability

The data presented in this study are available on request from the corresponding author.
